# Low bone morphogenic protein-2 in diabetes patients with peripheral neuropathy is a correlated risk factor for the development of Charcot arthropathy

**DOI:** 10.1007/s00592-025-02573-5

**Published:** 2025-09-17

**Authors:** Jean Cassuto, Agnetha Folestad, Martin Ålund, Susanne Asteberg, Jan Göthlin

**Affiliations:** 1 Orthopedic Research Unit, Department of Orthopedic Surgery, Sahlgrenska University Hospital, Mölndal, 43180 Sweden; 2 Department of Orthopedics, Frölunda Specialist Hospital, Västra Frölunda, Sweden; 3 Department of Radiology, Sahlgrenska University Hospital, Mölndal, Sweden; 4 Institution of Clinical Sciences, Göteborg University, Göteborg, Sweden

**Keywords:** Charcot neuroarthropathy, Neuropathy, Type 1 diabetes, Type 2 diabetes, Bone regeneration, Bone morphogenic proteins

## Abstract

**Aims:**

Diabetes patients with peripheral neuropathy run increased risk of developing Charcot arthropathy (Charcot), often associated with foot fractures. Bone morphogenic proteins (BMPs) are among the most important regulators of bone homeostasis and fracture repair but have not been investigated in the pathophysiology of Charcot. The current study aims to address this issue.

**Methods:**

Sixteen patients diagnosed with active Charcot were treated with total contact cast (TCC) and monitored during 24 months (M) with repeated plain radiographs and magnetic resonance imaging (MRI). Plasma was sampled at 9 occasions and analyzed for BMP-1, BMP-2, BMP-3, BMP-4, BMP-6, BMP-7 and BMP-9 as well as for basal laboratory data. Fifteen diabetes patients with peripheral neuropathy and fifteen healthy participants without diabetes served as controls.

**Results:**

All Charcot patients had pathologically low BMP-2 level at inclusion which remained suppressed throughout the 2-year follow-up as defined by being lower than 2 standard deviations (SD) of BMP-2 in healthy controls (*p* < 0.001) and in diabetes patients with neuropathy without Charcot (*p* < 0.002). BMP-2 did not differ between the control groups. BMP-7 in Charcot patients increased significantly 6–12 months following TCC treatment. Other BMPs showed no significant differences between the groups at any point during the follow-up.

**Conclusions:**

Low BMP-2 in diabetes patients with neuropathy is associated with increased risk of developing Charcot fractures due to the critical role of BMP-2 for the initiation of bone repair. BMP-7 appears to partly compensate for the lack of response by other osteogenic BMPs during fracture repair in Charcot patients.

**Supplementary Information:**

The online version contains supplementary material available at 10.1007/s00592-025-02573-5.

## Introduction

Early diagnosis of Charcot is often missed due to few or absent sensory symptoms from the affected foot which may lead to the development of foot fractures and distorsion [1]. Despite that the condition was first described in 1883, we know little of the mechanisms that trigger the pathophysiological and biomolecular events that trigger its onset and govern its progress and resolution [1]. Two predictive risk factors are currently linked to the pathogenesis of *Charcot*, i.e. type 1 or type 2 diabetes and peripheral neuropathy [1], both of which go with significantly increased fracture risk [2, 3]. Despite new insights into the complex biomolecular nature of bone repair [4, 5] and several mechanistic hypotheses having been presented over the years [1], we still search for answers as to why only 0.1-1% of diabetes patients will develop Charcot [6]. BMPs have been extensively investigated and shown to be a pivotal part of the biomolecular mechanisms that initiate fracture repair and regulate bone maintenance [7] with BMP-2 and BMP-7 having been approved by the federal drug administration (FDA) for clinical use in cartilage and bone tissue repair [8, 9]. Despite the importance of BMPs in fracture repair [7, 10, 11], there are at present no reports addressing their role in the pathogenesis of Charcot. In the current study we investigated the involvement of several osteogenic BMPs during the active bone repair phase of Charcot.

## Materials and methods

### Study design and population

The study was approved in 2007 by the Review Board of Västra Götalands Regionen (EPN 499-07) with additional approval 2010 (T762-10 ad 499-07). Healthy individuals and patients with diabetes neuropathy without Charcot that served as controls provided informed consent before participating in the study that has level of evidence II and complies with the STROBE-statement for observational studies [12]. Sixteen consecutive ambulatory men and women admitted to Sahlgrenska University Hospital/Mölndal with clinical signs of unilateral active Charcot arthropathy were included into the study. After having been informed that radiographs showed finalized bone healing, one patient interrupted participation before the 12 month radiograph and MRI but allowed for continued blood sampling and for data to be processed in the study. Inclusion of Charcot patients was based on medical history, clinical examination and radiographic findings and required the following criteria: (1) type 1 or type 2 diabetes with a duration of more than 1 year, (2) bilateral peripheral neuropathy as defined below, (3) clinical signs of active Charcot with hot/reddened/swollen foot and skin temperature in the affected foot being at least 2 °C higher than in the contralateral foot. Exclusion criteria were: (1) plantar ulcerations, (2) documented history of bone trauma or surgery during the past year prior to inclusion into the study, (3) ongoing immunosuppressive therapy or medications known to affect bone metabolism (e.g., bisphosphonates, denosumab). At inclusion, all Charcot patients received a non-weight-bearing total contact cast (TCC) which was repeatedly replaced as required due to changes in foot volume with increased or attenuated swelling. The offloading protocol was aided by crutches or wheelchair and was continued until the difference in skin temperature between the feet was 1 °C or less and no signs of redness and swelling were present for the past 30 days. TCC was at this point replaced by orthosis for partial weight-bearing. When full weight-bearing was allowed, patients received prescribed accommodative shoes. Two independent control groups were recruited into the study: (1) fifteen ambulatory individuals with diabetes type 1 or type 2 with peripheral neuropathy but no Charcot (2) fifteen healthy controls. None of the patients in the control groups had a history of medication for osteoporosis, documented history of joint/bone disease or bone trauma/surgery one year prior to inclusion. Neuropathy in Charcot patients and in neuropathic diabetes controls was defined by pathological albuminuria (> 300 mg/day), hypertension and declining renal function (Glomerular filtration rate < 60 mL/min/1.73m2).

### Skin sensitivity, skin temperature and toe pressure

Semmes-Weinstein monofilament test was used to measure skin sensitivity at 4 different locations on both feet [13]. The monofilament (10 g) was pressed against the skin and the patient´s ability or inability to feel the sensation upon buckling of the monofilament was registered. Neuropathy was present if three or more sites were insensate to the monofilament. Skin temperature was measured bilaterally at two locations on the dorsal foot, i.e. 5 cm distal of the ankle and 2 cm proximal of the mid toe, by means of a dual electrode thermometer (CIE 307, Taiwan). Toe blood pressure was measured bilaterally using a specially designed cuff [14]. Measurements of skin temperature and toe pressure were performed at inclusion, 1 week (W), 2 months (M), 4 M, 6 M, 8 M, 12 M, 18 M and 24 M postinclusion.

### Blood sampling and analysis of plasma biomarkers

Peripheral blood samples in Charcot patients were collected repeatedly for routine analysis (Hemoglobin, serum creatinine, C-reactive protein, blood sedimentation and HbA1c). Venous blood for cytokine analysis was drawn into EDTA tubes at inclusion, 1 W, 2 M, 4 M, 6 M, 8 M, 12 M, 18 M and 24 M postinclusion. Frequent routine laboratory monitoring of patients with diabetes neuropathy without Charcot that served as controls confirmed to us that no significant laboratory/clinical changes had occurred in this group during the duration of the study (Table 1). The latter observation formed the basis for our decision to use blood sampled for biomarker analysis at inclusion to serve as reference for biomarker levels in Charcot patients both at inclusion and at 24 months post-inclusion. Blood from healthy controls was sampled on a single occasion and used as reference at inclusion and at 2 years post-inclusion as we did not expect clinical and laboratory variables in the control groups to undergo significant changes during the short duration of the study. Plasma biomarkers were analyzed on a high-sensitivity and wide dynamic range platform from MesoScaleDiagnostics (Sector Imager 2400^®^; Rockville, Maryland, USA; for details see www.mesoscale.com*).* Matched pairs of antibodies, i.e., capture antibody (CA) and biotinylated detection antibody (DA) labeled with streptavidin SULFO-TAG^®^ were used for analysis of plasma biomarkers as specified below. Standard curves were created using human recombinant proteins (hRP). Plasma was mounted on uncoated standard plates from MSD (L15XA) and analyzed for BMP-1, BMP-2, BMP-3, BMP-4, BMP-6, BMP-7 and BMP-9 as specified below. BMP-1, CA: anti-human monoclonal rat IgG_2B_ antibody (Biotechne R&D Systems, cat.no.MAB1927), DA: produced in goats immunized with purified NSO-derived recombinant human BMP-1/PCP (procollagen C-proteinase) (aa121-730) and subsequently biotinylated (Biotechne R&D Systems, cat.no.BAF1927), hRP: mouse myeloma cell line, NSO-derived (Biotechne R&D Systems, cat.no.1927-ZN). BMP-2, CA: monoclonal mouse IgG_2B_ (Biotechne R&D Systems, cat.no.MAB3551), DA: biotinylated monoclonal mouse IgG_1_ antibody (Biotechne R&D Systems, cat.no.BAM3552), hRP: E-coli-derived (Prospecbio cat.no.CYT-261). BMP-3, CA: monoclonal mouse IgG_2B_ (Biotechne R&D Systems, cat.no.MAB1876), DA: biotinylated antigen affinity-purified polyclonal goat IgG antibody (Biotechne R&D Systems, cat.no.BAF113), hRP: E-coli-derived (Biotechne R&D Systems, cat.no.113-BP). BMP-4, CA: monoclonal mouse IgG_2B_ (Biotechne R&D Systems, cat.no.MAB7571), DA: biotinylated monoclonal mouse IgG_1_ antibody (Biotechne R&D Systems, cat.no.BAM7572), hRP: E-coli-derived (Prospecbio cat.no.CYT-361). BMP-6, CA: monoclonal mouse IgG_2B_ (Biotechne R&D Systems, cat.no.MAB507),

**Table 1:** Clinical and demographic data in Charcot patients, neuropathic diabetes controls, and healthy controls at inclusion and 2Y post-inclusion

	Charcot arthropathy at inclusion (*n* = 16)	Charcot arthropathy at 2Y (*n* = 15)	Neuropathic diabetes controls at inclusion (*n* = 15)	Neuropathic Diabetes controls at 2Y (*n* = 15)	Healthy controls (*n* = 15)
Age (years)	68 ± 2		57 ± 3		69 ± 3
Females	4	3	6	6	9
Males	12	12	9	9	6
Diabetes type 1/2	4/12	3/12	5/10	5/10	–
Diabetes duration (years)	15 ± 3	–	13 ± 1	–	–
Debut of foot symptoms (weeks) (swelling, redness, increased warmth)	10 ± 2	–	–	–	–
Warm/red/swollen foot	16/10/16	14/8/14	–	–	–
Skin temperature (Celcius)					
Charcot foot	32.1 ± 0.8***	29.1 ± 0.8	Left foot 29.7 ± 0.4	Left foot 29.1 ± 0.3	–
Contralateral foot	28.7 ± 0.6	29.3 ± 0.5	Right foot 30.0 ± 0.4	Right foot 29.8 ± 0.4	–
Pain at rest	2	2	–	–	–
Pain at weight bearing	12	12	–	–	
Insensate neuropathic foot					
Charcot foot	15	15	Left foot 15	Left foot 15	–
Contralateral foot	14	14	Right foot 15	Right foot 15	–
Broken Meary line	10	10	–	–	–
Systolic blood pressure (mmHg)	145 ± 6	149 ± 4	128 ± 3	–	132 ± 3
Diastolic blood pressure (mmHg)	80 ± 3	77 ± 2	76 ± 1	–	79 ± 2
Arterial toe pressure (mmHg)					
Charcot foot	128 ± 10	128 ± 6	Left foot 117 ± 3	Left foot 119 ± 3	–
Contralateral foot	125 ± 7	125 ± 6	Right foot 117 ± 4	Right foot 117 ± 4	–
Total contact cast (months)		10.9 ± 1.2	–	–	–
Nephropathy	2	2	2	2	–
C-reactive protein (mg/L)	10 ± 3	7.3 ± 2	3.9 ± 0,9	3.8 ± 0,8	3.1 ± 0,6
Blood sedimentation (mm)	30 ± 6^§,^**	26 ± 6	12 ± 2	10 ± 2	8 ± 3
Creatinine (µmol/L)	96 ± 11	100 ± 12	77 ± 5	79 ± 7	82 ± 7
HbA1c (mmol/mol)	62 ± 3	65 ± 3	59 ± 2	56 ± 2	–
HbA1c (%)	7.8 ± 0.3	8.0 ± 0.2	7.6 ± 0.2	7.3 ± 0.2	–

***p < 0.001 Charcot affected foot versus contralateral foot at inclusion

**p = 0.001 Charcot versus healthy controls at inclusion

^§^*p* = 0.003 Charcot versus Neuropathic diabetes controls at inclusion

All other differences were not significant. Mean ± SEM

DA: biotinylated antigen affinity-purified polyclonal goat IgG antibody (Biotechne R&D Systems, cat.no.BAF507), hRP: E-coli-derived (Prospecbio cat.no.CYT-754). BMP-7, CA: antigen affinity-purified anti-human polyclonal rabbit IgG antibody (Peprotech, cat.no.500-P198), DA: biotinylated antigen affinity-purified polyclonal rabbit IgG antibody (Peprotech, cat.no.500-P198Bt), hRP: E-coli-derived (Prospecbio cat.no.CYT-333). BMP-9, CA: monoclonal mouse IgG_2B_ (Biotechne R&D Systems, cat.no.MAB3209), DA: biotinylated antigen affinity-purified polyclonal goat IgG antibody (Biotechne R&D Systems, cat.no.BAF3209), hRP: Chinese hamster ovary cell line, CHO-derived (Biotechne R&D Systems, cat.no.3209-BP). Antibodies were optimized by checkerboard titrations and control of standard curves. Inter-assay variations were < 5%.

### Radiographs and MRI

Radiographs of both feet were performed in supine position with dorsoplantar, oblique and lateral projections as well as weight-bearing in frontal and lateral projections. Examinations followed a preset schedule starting at inclusion, a week after inclusion and subsequently at 6 M, 12 M, 18 M and 24 M post-inclusion. Magnetic resonance imaging (MRI) was done in accordance with the latter schedule and was performed on a 1.5 T Magnetom^®^ Symphony (Siemens) with supine patient and feet first into the gantry. All examinations were performed with a head-neck surface coil and feet in flexed position. Each foot was examined by use of 4 sequences without intravenous contrast medium: T1, T2, T2 3D and STIR sequences in sagittal, transverse and coronary positions. Intravenous contrast was followed by T1-sequences in transverse and sagittal projections. MRI was analyzed for disease activity by presence of bone marrow edema and soft tissue edema (Table 2).

**Table 2:** Staging of radiographic changes in the Charcot foot as described by Eichenholz [6] and based on weight-bearing radiographic examinations of the diseased foot

Radiographs	Inclusion (*n* = 16)	1 week (*n* = 16)	6 months (*n* = 16)	12 months (*n* = 15)	18 months (*n* = 15)	24 months (*n* = 15)
Stage 0	2	0	0	0	0	0
Stage 1	6	8	5	2	2	0
Stage 2	8	8	10	9	3	1
Stage 3	0	0	1	4	10	14
MRI	Inclusion	1 week	6 months	12 months	18 months	24 months
Bone marrow edema	**-**	16	14	9	6	1
Soft tissue edema	-	16	14	7	5	3

MRI was analyzed for bone marrow edema and/or soft tissue edema in the Charcot foot

**Table 3:** Comparison between Charcot arthropathy, neuropathic diabetes controls and healthy individuals at inclusion and at 2Y post-inclusion were done by one-way repeated measures ANOVA

Unit	At inclusion to the study				At 2Y post-inclusion			
pg/ml	Charcot	Diabetes controls	Healthy	P-value	Charcot	Diabetes controls	Healthy	P-value
BMP-1	2156 ± 325	2452 ± 254	2401 ± 222	0.477	3843 ± 612	2452 ± 254	2401 ± 222	0.252
BMP-2	15 ± 1.4	82 ± 19***	65 ± 12^§§^	***p < 0.001 ^§§^*p* = 0.004	20 ± 3	82 ± 19***	65 ± 12^§§^	***p < 0.001 ^§§^*p* = 0.003
BMP-3	806 ± 215	1639 ± 280	1114 ± 185	0.099	1688 ± 309	1639 ± 280	1114 ± 185	0.356
BMP-4	315 ± 49	344 ± 53	313 ± 52	0.461	283 ± 42	344 ± 53	313 ± 52	0.334
BMP-6	189 ± 25	294 ± 42	270 ± 34	0.056	245 ± 41	294 ± 42	270 ± 34	0.084
BMP-7	80 ± 15	130 ± 18	207 ± 31	0.252	143 ± 27	130 ± 18	207 ± 31	0.055
BMP-9	345 ± 36	387 ± 34	339 ± 23	0.475	407 ± 51	387 ± 34	339 ± 23	0.544

***p < 0.001 Charcot versus healthy at inclusion and 2Y post-inclusion

^§§^Charcot versus neuropathic diabetes controls at inclusion and 2Y post-inclusion

Mean ± SEM

Staging of disease characteristics was based on clinical observations and plain radiographs, as described by Eichenholz [6], and comprised of 4 stages:* stage 0*: warm, reddened, swollen foot and normal radiographs,* stage 1*: swollen, inflamed foot and radiographic evidence of macro-fractures, bone fragmentation, bone debris absorption, articular diastases,* stage 2*: bone repair and remodeling and callus formation,* stage 3*: finalized bone healing (Table 2).

### Statistical methods

Data are presented as mean ± SEM or mean ± SD. One-way repeated measures analysis of variance (ANOVA) with post hoc Holm-Śidak test was used for comparisons of differences in demographic and laboratory data between the three groups of the study (Table 1) with Charcot representing a continuous dependent variable (time) whereas the independent categorical groups are represented by diabetes neuropathy patients without Charcot and healthy individuals. Normality was assessed by Shapiro-Wilk and Kolmogorov-Smirnov tests. Equal variance was tested by use of the Brown-Forsythe test. Log transformation was used to normalize data when necessary. Differences in biomarker levels between Charcot and the two control groups were analyzed by one-sample t-test when normality was achieved and otherwise by signed rank test which was also used for comparison of biomarker levels within the Charcot group (Table 3). One-sample t-test was used to compare the cumulative average (mean) of BMP-2 in Charcot patients i.e. the sum of all values at each position divided by the total number of observations, i.e. at inclusion, 1 W, 2 M, 4 M, 6 M, 8 M, 12 M, 18 M, 24 M versus all the individual values of neuropathic diabetes controls and healthy individuals.

## Results

**Fig. 1:**
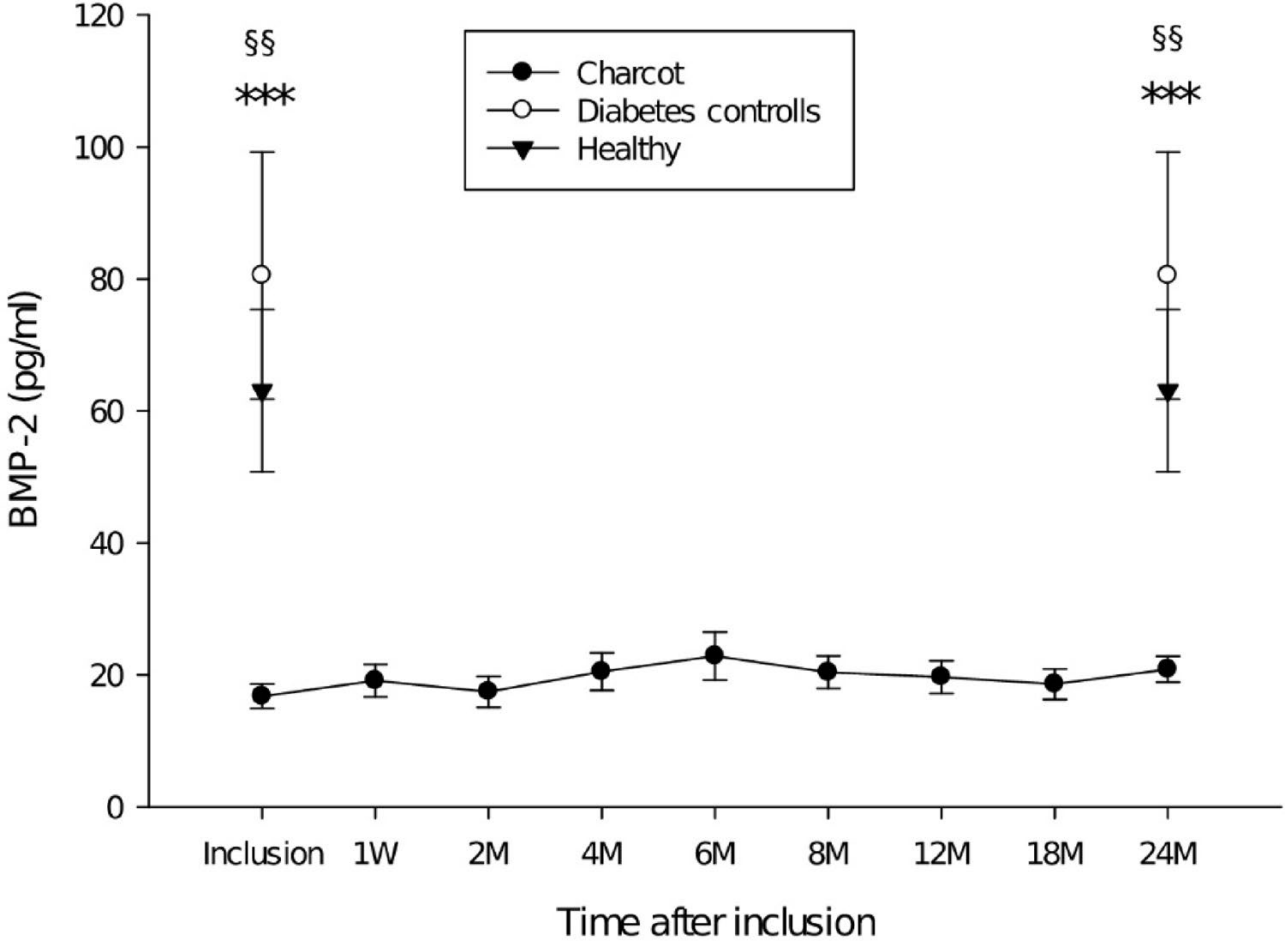
Plasma BMP-2, ***p < 0.001 Charcot vs. healthy at inclusion and at 2Y postinclusion, ^§§^ Charcot vs. neuropathic diabetes controls at inclusion (*p* = 0.004) and at 2Y postinclusion (*p* = 0.003). Neuropathic diabetes controls vs. healthy showed no significant differences. Mean ± SEM

**Fig. 2:**
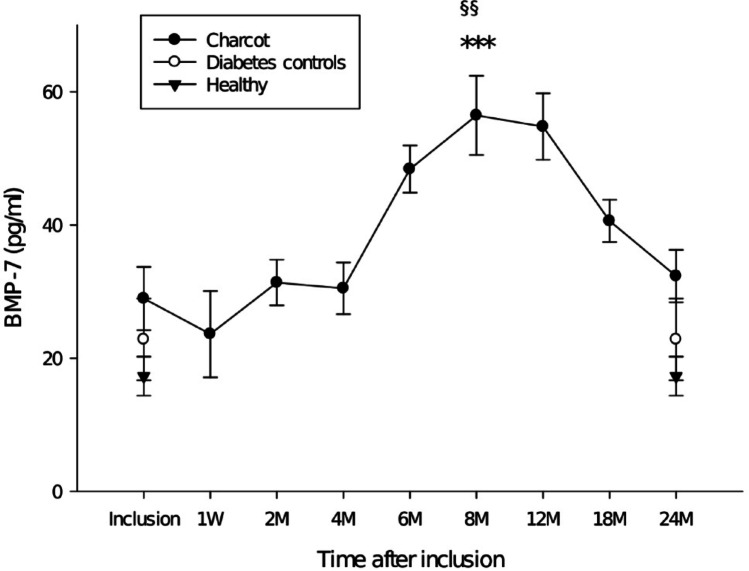
Plasma BMP-7, *** *p* < 0.001 Charcot vs. healthy at inclusion and at 2Y post-inclusion, §§ *p* = 0.003 Charcot vs. neuropathic diabetes controls at inclusion and *p* = 0.005 at 2Y post-inclusion. Other differences were not significant. Mean ± SEM

Clinical and demographic data are presented in Table 1. Data from plain radiographs and MRI are presented in Table 2. Radiographs showed that all Charcot patients had foot fractures on radiographs taken one week post-inclusion and that all, except one patient, had reached a stage of final bone healing at 2Y post-inclusion. MRI showed all Charcot patients to have bone marrow edema at 1 week post-inclusion as a sign of active disease in the affected foot with all, but one patient, having reached a resolution phase with complete regression of bone edema at 2Y post-inclusion. BMP-1, BMP-3, BMP-4, BMP-6 and BMP-9 showed no significant differences between Charcot patients, neuropathic diabetes controls and healthy individuals at any point during the follow-up with levels of significance between the groups at inclusion and at 2Y post-inclusion being presented in Table 2. The mean ± SD of BMP-2 at inclusion was: Charcot 16 ± 7 pg/ml, neuropathic diabetes controls 80 ± 133 pg/ml, healthy individuals 63 ± 71 pg/ml. The number of patients with BMP-2 below 30 pg/ml at inclusion was for Charcot 16 out of 16 (16/16, 100%), for neuropathic diabetes controls (6/16, 37%) and for healthy individuals (2/16, 13%). Plasma BMP-2 was significantly lower in Charcot versus neuropathic diabetes controls at inclusion (*p* = 0.004) and at 2Y post-inclusion (*p* = 0.003). Plasma BMP-2 was significantly lower in Charcot versus healthy individuals at inclusion (*p* < 0.001) and at 2 Y post-inclusion (*p* < 0.001). Differences between the accumulated mean of BMP-2 values in Charcot patients were significantly lower than BMP-2 in neuropathic diabetes controls (*p* < 0.001) and healthy (*p* < 0.001). Differences between neuropathic diabetes controls and healthy were not significant at any point during the follow-up (Fig. 1). BMP-2 in Charcot patients did not differ significantly by gender. BMP-7 in Charcot patients did not differ significantly from neuropathic diabetes controls or healthy individuals at inclusion but increased to a peak at 8 M post-inclusion which was significantly above healthy individuals (*p* < 0.001) and neuropathic diabetes controls (*p* = 0.003) but returned to a level not significantly different from controls at 2Y post-inclusion. No significant differences were seen between any of the study groups with regard to other BMPs of the current study (Fig. 2).

## Discussion

**Fig. 3:**
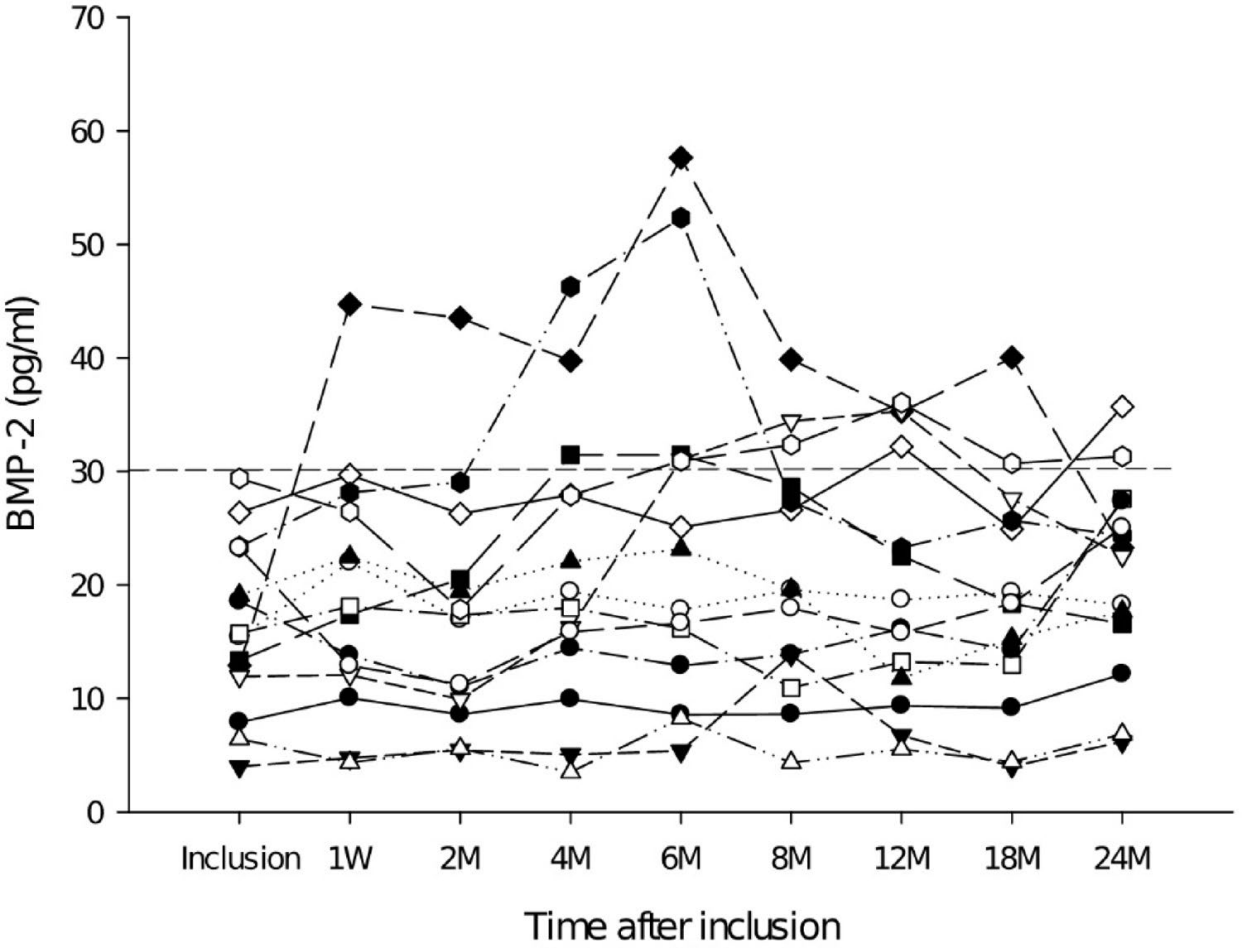
BMP-2 trends in individual Charcot patients during the entire duration of the study. The horizontal dotted line marks 30 pg/ml, below which BMP-2 is defined as low

The most notable finding of the current study was that BMP-2 in Charcot patients was more than 2 SD below the mean of BMP-2 in healthy and neuropathic diabetes controls. Since only neuropathic diabetes controls with BMP-2 below 30 pg/ml developed Charcot, it could be postulated that neuropathic diabetes controls with plasma BMP-2 below 30 pg/ml are at increased risk of developing Charcot arthropathy than neuropathic diabetes controls with BMP-2 above that level. The latter would be supported by the current study showing that 100% of Charcot patients had a BMP-2 values < 30 pg/ml as opposed to neuropathic diabetes controls (37%) and healthy (13%). The incidence and prevalence of Charcot varies from 0.1 to 0.4% in people with diabetes but increases to 35% in patients with peripheral neuropathy [6]. The latter aligns with 37% of neuropathic diabetes controls of the current study having BMP-2 levels below 30 pg/ml thus being associated with the development of Charcot. A notable observation of the study was that all Charcot patients had low BMP-2 (< 30pg/ml) at inclusion and had all developed foot fractures on radiographs taken one week post-inclusion. It could be argued that the significantly suppressed level of BMP-2 in all the patients with active Charcot at inclusion into the study is inherent to diabetes patients that develop Charcot arthropathy. However, we had no measurements of BMP-2 in Charcot patients prior to their arrival at our hospital with active disease which did not allow us to decide whether the low BMP-2 level is inherent to neuropathic diabetes control patients that ultimately develop Charcot or whether they had normal levels of BMP-2 before disease onset and acquired low levels at the onset of Charcot pathology. Presentation of individual BMP-2 levels in Charcot patients during the length of the study (Fig. 3) revealed that two patients were able to respond strongly after TCC treatment by significantly increasing their BMP-2 levels during the phase of bone healing before returning to levels below 30 pg/ml. The latter 2 patients that deviated from the bulk of the Charcot population are particularly interesting as they may represent the normal variation within the Charcot population or offer a glimpse into what would be the expected response by BMP-2 during bone repair in neuropathic diabetes patients without Charcot. Although the lack of BMP-2 is normally associated with lethality, heterozygous mice lacking limb-specific BMP-2 expression showed a dose-dependent decrease in bone mineral density (BMD) relative to healthy littermates but did not develop spontaneous fractures at ½ the amount of BMP-2 of healthy littermates [10]. However, BMP-2 level in the current Charcot population was less than a 1/5 of the mean in neuropathic diabetes controls which is likely to cause a more pronounced decrease of BMD and increase the likelihood of spontaneous foot fractures upon weight-bearing [3, 10]. The latter is supported by a study showing that patients with senile osteoporotic fractures had significantly lower BMD and serum BMP-2 than healthy controls as well as significantly prolonged duration of fracture healing [15]. The importance of BMP-2 for endochondral bone repair has been established by several experimental studies showing it to recruit and regulate the fate of skeletal progenitor cells towards the chondrogenic and osteogenic lineages [16]. In a study on transgenic mice with limb specific inactivation of BMP-2, the authors showed normal development of the limb skeleton before birth but a significant retardation of bone formation in cartilaginous ossification centers as early as 1 week post-partum in the BMP-2-lacking limbs [10]. This led to multiple weight-bearing fractures that could not heal due to total inability to produce callus and bridge the fracture gap [10]. Molecular analysis of the fracture site revealed that mesenchymal stem cells (MSCs), although present, failed to differentiate into chondrocytes that form the chondrogenic callus required for endochondral bone repair to initiate [10].

BMP-2 has also been reported to dose-dependently increase chondrocyte hypertrophy and mineralization, a mandatory step for the transformation of the cartilaginous callus into the bony callus [17] and its subsequent remodeling into bone [16]. In a recent study in otherwise healthy hip implant patients there was a significant increase by BMP-1, BMP-2, BMP-3, BMP-7 and BMP-9 during the early phases of endochondral bone repair and by BMP-4 during the phase of coupled bone remodeling [11]. This is in stark contrast to the Charcot population of the current study showing only BMP-7 to increase significantly during the phase of bone repair whereas all other BMPs of the study remained at the level of neuropathic diabetes controls and healthy individuals throughout the follow-up. However, despite the lack of response by several osteogenic BMPs, all Charcot patients, but one, were able to reach the final stage of bone repair which could, at least in part, be attributed to a significant redundancy among BMPs that enables them to compensate for lack of response by other family members [18]. Thus, the multifaceted pro-osteogenic role of BMP-7 during endochondral fracture repair includes the facilitation of MSC differentiation into chondrocytes that form the initial cartilagenous callus [19], promotion of cartilage remodeling through its stimulatory effects on matrix metalloproteinases (MMPs) [20] and *a* potent induction of osteoblast formation [19, 21] while at the same time downregulating the formation and mineralization of the hypertrophic cartilage which is mandatory for endochondral bone repair to proceed from the cartilagenous phase to the bony phase [17]. BMP-7 does also have anti-inflammatory effects [22] that contribute to the resolution of inflammation which is critical for the transformation of cartilage into bone [23]. However, sustained increase by BMP-7 during bone repair requires the activation of BMP-2 as was shown in a study in mice lacking limb specific BMP-2 and showing no production of BMP-7 due to failure of MSC to differentiate into chondrocytes and osteoblasts [10]. In contrast, heterozygotic mice with reduced BMP-2 did produce BMP-7 that allowed bone repair to progress [10]. The latter study suggests that despite suppressed BMP-2 in Charcot patients and despite lack of response by other BMPs, BMP-7 with the likely assistance of other osteogenic factors enabled bone repair to proceed to completion as was shown by radiographic data of Charcot patients (Table 1).

## Conclusions

The small cohorts of participants in the current study may not allow us to conclude that low BMP-2 in neuropathic diabetes patients is *per* se a predictive factor or a risk factor for the development of Charcot but lend strong support to BMP-2 deficiency being associated with the pathogenesis of Charcot. Such a conclusion would be supported by the critical role of BMP-2 in the maintenance of the normal skeleton and in the initialization of bone repair [7]. We cannot at present rule out other, yet unknown, factors that besides low BMP-2 are required for the pathological processes of Charcot to initiate. Nonetheless, recombinant human (rh) BMP-2 has been approved by FDA for clinical use as an alternative to autograft bone [24] and has been shown to significantly improve bone healing [25–28]. Off-label use of rhBMP-2 in foot and ankle fusions has yielded good results with few side effects [26–28]. In one of the latter studies [26], rhBMP-2 was shown to improve ankle arthrodesis in all subgroups (Charcot, diabetes, infection and smokers), with particular emphasis made on improvement in Charcot patients, thus supporting rhBMP-2 as a viable alternative to bone grafts that should be considered as a potential adjunct in the treatment of Charcot patients with particularly severe and complicated foot fractures that require surgical treatment, not least when considering their deficient BMP-2 levels.

## Supplementary Information

Below is the link to the electronic supplementary material.

Supplementary Material 1

Supplementary Material 2

Supplementary Material 3
