# Supplementary material for: Low bone morphogenic protein-2 in diabetes patients with peripheral neuropathy is a correlated risk factor for the development of Charcot arthropathy
Source: Acta Diabetol. 2025 Sep 17;63(1):123–31. doi: 10.1007/s00592-025-02573-5 (PMC12847135; doi:10.1007/s00592-025-02573-5)
Supplement: Supplementary file 1 — Supplementary Material 1 [file 592_2025_2573_MOESM1_ESM.docx]

April 3, 2025

Editor-in-Chief

Massimo Federici

Dear Editor,

Charcot neuro-arthropathy affects primarily diabetic individuals, type 1 and 2 alike, and is a serious complication to diabetes characterized by bone destruction in a foot leading to disability and a common cause of foot amputations. Despite being diagnosed and described more than a century ago, the mechanisms triggering and sustaining the pathological changes in the affected foot remain largely unknown. This has limited the spectrum of treatment alternatives to unloading the diseased foot in total contact cast with no means of aiming at specific key factors in the pathophysiology, as has been the case for several other bone diseases such as RA and osteoarthritis. Better understanding of the mechanisms guiding a wide range of processes regulating the maintenance and repair of bone tissues, has prompted increasing number of reviews to discuss possible molecular mechanisms in the pathophysiology of Charcot arthropathy, although there is very little data to sustain the proposed hypotheses. In view of extensive research on the role of bone morphogenic proteins (BMPs) that has highlighted their critical role in the regulation of bone mineral density and fracture repair and a recent longitudinal clinical study presented by us on the importance of for bone repair (Cassuto, J et al. (2023) The importance of BMPs and TGF-βs for endochondral bone repair-a longitudinal study in hip arthroplasty patients. Bone reports: vol.19, 101723), we are unaware of any study investigating the role of BMPs in the pathophysiology of Charcot.

In this observational clinical study we investigated the role of several osteogenic BMPs in patients having been admitted to our hospital on the suspicion of having acute Charcot neuro-arthropathy. When included into the study by fulfilling several criteria, patients were monitored repeatedly by blood samples for BMP analysis, plain radiographs and MRI during 24 months.

I am aware of the fact that although the current study involves diabetes patients with diabetes complications it may be beyond the scope or ability of the journal to find suitable reviewers that are knowledgeable in the field of fracture repair. If the journal is unable to find reviewers please do not hesitate to let me know and I will retract the manuscript and I assure you that this will NOT affect my future work with the journal as a reviewer.

Kind regards,

J Cassuto, MD, PhD

Manuscript text: MS Word 2010, Graphics: Sigma Plot 12.5, References: Endnote X7
